# The Changing Landscape of Legume Products Available in Australian Supermarkets

**DOI:** 10.3390/nu13093226

**Published:** 2021-09-16

**Authors:** Dale Bielefeld, Jaimee Hughes, Sara Grafenauer

**Affiliations:** 1 Positive Central, Redfern Health Centre, Sydney Local Health District, Sydney, NSW 2000, Australia; dbie0493@uni.sydney.edu.au; 2 Grains & Legumes Nutrition Council, 1 Rivett Road, North Ryde, NSW 2113, Australia; j.hughes@glnc.org.au; 3 School of Medicine, University of Wollongong, Northfields Avenue, Wollongong, NSW 2522, Australia

**Keywords:** legumes, pulses, nutrition information, plant protein, sustainability

## Abstract

Evidence supports regular dietary inclusion of legumes due to their positive effects on both human and planetary health. Intake within Australia is suboptimal, with consumer data suggesting that an inability to integrate legumes into usual dietary patterns is a barrier to consumption. This places the food industry in a unique position to offer Australians the ability to incorporate legumes into usual dietary patterns via innovative new products. The aim of this study was to explore the legume category and compare nutrition product data and the use of nutrition and health claims between 2019 and 2021. An audit of legume products from four major metropolitan Sydney supermarkets (Aldi, Coles, IGA, Woolworths) collected ingredient lists, nutrition information and on-pack claims for baked beans, legume dips, legume flours, legume snacks (including subcategories of legume chips and whole legume snacks), canned legumes, dried legumes, frozen legumes, and pulse pasta. The total number of legume products available on the market nearly doubled from 2019 (*n* = 312) to 2021 (*n* = 610); this was driven by traditional plain canned and dried legumes and some new and convenient options, particularly snacks (legume chips) where the largest growth occurred. Of all legume products (*n* = 610), 82% met the Nutrient Profiling Scoring Criteria, 86.8% were at least a source of dietary fibre, and 55.9% were at least a source of protein. Nutrition content claims relating to dietary fibre, gluten free and protein more than doubled since 2019, with each featuring on over one third of the products identified in 2021. Vegan/vegetarian on-pack claims more than doubled since 2019, and claims related to the term plant-based/plant protein and environmental sustainability emerged on packs in 2021. By addressing barriers to consumption, such as lack of time and knowledge on how to prepare legumes, innovative legume products may help influence future consumption patterns.

## 1. Introduction

Legumes, such as chickpeas (*Cicer arietinum*), beans (*Phaseolus vulgaris*), peas (*Pisum sativum*), lentils (*Lens culinaris*) and dried pulses, are an excellent dietary source of plant protein, dietary fibre and minerals [1]. Regular consumption of legumes contributes to improved dietary quality and nutrient density [2], with regular intake associated with improved markers of metabolic health, weight management, reduced risk of coronary heart disease (CHD) and reduced risk of all-cause mortality [3,4,5,6,7]. Due to their nutritious and ecologically sustainable qualities, the Food and Agriculture Organisation of the United Nations (FAO) have recognised legumes as a key pillar in addressing the sustainability of agricultural and food systems as well as food security [1]. Recognised for their nitrogen-fixing properties, legume crops facilitate a regenerative effect, improving soil fertility and reducing greenhouse gas emissions via a reduction in the use of fertilisers [1,8]. Environmental sustainability and human health are both intricately linked to diet [9]. A substantial body of evidence indicates that dietary patterns rich in plant foods, including an emphasis on legumes, consumed in preference to animal-sourced foods hold the key to optimising human and planetary health [9]. Despite this, the integration of legumes into the dietary patterns of Western-based countries, such as Australia, presents a challenge [1,10]. 

National healthy eating guidelines globally recommend the consumption of legumes in variable quantities [11,12,13,14,15], with the Australian Dietary Guidelines (ADG) encouraging consumption as part of the ‘vegetables’ food group (1 serve = 75 g) and the ‘lean meat and alternatives’ food group (1 serve = 150 g) [13]. Variability exists within the evidence base, and a unified daily target is lacking [16]; however, the Eat-Lancet Planetary Health Diet suggests a scientific target of 50 g/day of legumes (dry beans, lentils and peas) (range 0–100 g/day) [9]. Irrespective of the discord surrounding an ideal daily target, legume consumption within Australia is inadequate. A secondary analysis of the 2011–2012 National Nutrition Physical Activity Survey (NNPAS), found that only 7.9% of the population sampled had consumed legumes the day prior to the survey, with an average serving size of 100 g [17]. The median intake is estimated to be 4 g/day, with 44% of a population of Australians sampled reportedly being non-consumers of legumes (unpublished data). On a global scale, the average per capita intake has remained largely unchanged in the previous three decades, at 21 g/day [8]. Global legume intake would need to increase by more than 100% to meet the reference intake as outlined in the Eat-Lancet Planetary Health Diet [9].

To influence population dietary consumption of legumes, consumer preferences, drivers and barriers to consumption must be understood [18]. While some consumers are increasingly making the conscious decision to select foods that are sustainably grown and produced [18], Australian consumer data suggests that there are several negating factors preventing increased legume consumption [19]. Barriers to consumption are reported to commonly arise from a lack of culinary knowledge and/or skills, the time (perceived and/or actual) required for preparation, the perception of legumes being inconvenient to prepare and an aversion to the taste and/or texture of legumes [19,20]. This has placed the food industry in a unique position, with the opportunity to enhance population consumption by offering innovative and convenient legume products. A greater understanding of the types of legume products that are available will assist in determining whether legume products can complement overall legume intake by offering additional opportunities for consumption. This study aimed to explore the legume category and compare product numbers and the use of nutrition and health claims between 2019 and 2021.

## 2. Materials and Methods

A comprehensive audit of commercially available legume products was conducted in February 2019 and 2021 across four major supermarkets within metropolitan Sydney, Australia. The data collection methodology replicated that previously published by Grafenauer et al. (2018) [21] and targeted four retail supermarket chains; Aldi, Coles, Independent Grocers of Australia (IGA) and Woolworths, which together represent 79.2% of the Australian grocery market share. This methodology is consistent with results reported by Figueira et al. (2019) [19], as 95% of surveyed respondents reported to purchase legumes for home use from supermarkets. 

### 2.1. Eligibility and Exclusion Criteria

All legume products were assigned to one of eight categories, as outlined in Table 1 below, including baked beans, legume dips, legume flours, legume snacks, legumes canned, legumes dried, legumes frozen and pulse pasta. 

Products excluded from the data collection process were those derived predominantly from peanuts (e.g., peanut butter), as despite botanical classification within the *Leguminosae* family, this oil-seed legume carries a distinctly different culinary classification to that of other legumes [22]. The exclusion of peanuts parallels the classification of peanuts within the ‘seed and nut’ category, rather than ‘legumes and pulses’, as per the 2011–2012 NNPAS [23]. Legume dips packaged with crackers were captured within the audit, however only the legume-containing component was included in the analysis (*n* = 13). Combination legume products, for example, canned tuna and beans, mixed frozen peas and corn and ready-made meals containing legumes were deemed to be outside the scope of the current analysis and were therefore excluded from data collection. Legume-containing products marketed as meat alternatives (e.g., plant-based burgers), were also excluded from the data collection process as this category has been reviewed separately [24]. 

### 2.2. Ethics Approval

This study was exempt from requiring ethics approval given the analysis focused solely on food products; however, permission for data collection in-store was obtained from supermarket store managers.

### 2.3. Data Collection and Analysis

Smartphones were used to photograph the following information for each product: Ingredient list, Nutrition Information Panel (NIP), health and nutrition-related claims and Health Star Rating (HSR). A data extraction form was created in Microsoft^®^ Excel (Redmond, WA, USA), where the data (collected in both 2019 and 2021) was transcribed from photographs and collated for analysis according to the product category classifications outlined in Table 1. Data was confirmed by a second, independent reviewer to identify and amend inconsistencies or errors and cross-checked via the Mintel New Product Data Base. In addition to the in-store audit, a supplementary internet search was conducted via retailer websites and websites of manufacturers that were identified during in-store data collection. Although several products were available in numerous supermarket chains, each product was only recorded once, and data was screened to remove duplicates. 

The Food Standards Australia New Zealand (FSANZ) Nutrient Profiling Scoring Criterion (NPSC) was calculated for all products identified in the 2021 data set. The NPSC is a nutrient profiling method used to determine whether a food is eligible to carry general-level and high-level health claims, based on its nutrient profile [25]. The NPSC algorithm considers both positive nutrients/food components (e.g., dietary fibre, protein, fruit, vegetable, nut and legume content) and risk nutrients (e.g., energy, sugar, sodium and saturated fat). For products outlined in Table 1, the final NPSC score must be less than four. 

On-pack claims were classified as either nutrition content, general-level health claims or high-level health claims as per Standard 1.2.7 of the Australia New Zealand Food Standards Code (FSC) [26]. Other claims not covered by Standard 1.2.7 were also recorded (e.g., suitable for vegetarians/vegans, no artificial colours, flavours or preservatives). Data collected in 2021 was also assessed for eligibility to make nutrition content claims, in line with Standard 1.2.7 of the FSC. Australian labelling requirements do not require dietary fibre to be declared in the NIP unless a relevant on-pack nutrition content claim has been made [26]. Products that did not declare the dietary fibre content on the pack, were excluded from the dietary fibre claim eligibility calculation as to not skew the data and provide a misleading representation of the categories. 

Descriptive analyses were conducted with the aid of Microsoft^®^ Excel Version 16.50 (Redmond, WA, USA) to determine the number (*n*) and relative (%) change over time for each product category. 

## 3. Results

As outlined in Table 2, a total of 610 products were identified in the 2021 audit, including legume snacks (*n* = 140) (comprised of legume chips (*n* = 96), whole roasted legumes, savoury (*n* = 37) and whole roasted legumes, sweet (*n* = 7)), canned legumes (*n* = 154), legume dips (*n* = 107), dried legumes (*n* = 92), baked beans (*n* = 47), frozen legumes (*n* = 32), pulse pasta (*n* = 32) and legume flours (*n* = 6). The 2021 audit revealed a 95.5% increase in the number of products as well as an increase among all defined product categories compared to 2019. The legume snacks category experienced the greatest growth in the number of products, specifically legume chips which increased by an additional 75 products, followed by canned legumes (*n* = 72), dried legumes (*n* = 63), and legumes dips (*n* = 31). Legume chips (357% increase), whole legumes, sweet (250% increase), and dried legumes (217% increase) experienced the greatest change over time. 

A total of 95 food manufacturers/importers were represented across products identified in 2021, with Woolworths (NSW, Australia), Coles (VIC, Australia) and H.J. Heinz Company Australia Ltd. (VIC, Australia) being the top three, which were responsible for a collective 16.1% of products. The total number of manufacturers/importers increased 72.7% compared to 2019 (*n* = 55).

### 3.1. Legume Varieties

As displayed in Table 3, a total of 22 different varieties of legumes were identified among products in 2021, a relative increase of 22.2% over time. The largest increase in the number of products over time occurred among beans (*n* = 100 additional products), followed by chickpeas (*n* = 76) and mixed variety products (a combination of beans, chickpeas, peas, lentils and/or lupin) (*n* = 50). Within the beans category, edamame/soybeans experienced the greatest increase in the number of products over time (*n* = 18 additional products), followed by black beans/black turtle beans (*n* = 17) and kidney beans (*n* = 15). Legume types with the greatest relative change over time included mung beans (an increase of 600%), edamame/soybeans (an increase of 300%) and adzuki beans (an increase of 300%).

### 3.2. On-Pack Claim Eligibility and NPSC

Product eligibility for nutrition content claims varied among product categories, as outlined in Table 4. Most canned legumes (63.6%), dried legumes (94.6%), baked beans (95.7%), frozen legumes (50%), pulse pasta products (100%) and legumes flours (100%) were at least a source of protein. Similar results were found for dietary fibre claim eligibility, with 86.8% (361/416) of all products that declared the dietary fibre content on the pack eligible to carry at least a source of fibre claim. Most legume flours, dried legumes, frozen legumes and pulse pasta products were considered low in sodium, however only 4.7% of legume dips, 6.4% of baked beans, and 7.1% of legume snacks, were eligible to carry a low sodium claim. 

As presented in Table 4, all canned legumes (*n* = 154), dried legumes (*n* = 32), baked beans (*n* = 47), pulse pasta (*n* = 32) and legume flours (*n* = 6) categories passed the NPSC and were considered a healthier choice. Of the legume dips, 58.9% passed the NPSC with a median legume content (per 100 g) of 60 g (range 10–86 g). More than half of legume snacks passed the NPSC with an overall median legume content (per 100 g) of 43 g (5–98 g), 87 g (15–100 g) and 43 g (43–50 g) for legume chips, whole legumes, savoury and whole legumes, sweet, respectively. 

### 3.3. On-Pack Claims

Table 5 outlines the number and proportion of legume products that displayed nutrition content claims, general-level health claims, high-level health claims, and other claims. Nutrition content claims related to dietary fibre, gluten free and protein more than doubled since 2019, with each featuring on over one third of the products identified in 2021. A total of 14 different products displayed general-level health claims in 2021, increasing from just six products in 2019. Protein-related general-level health claims increased four-fold, while the number of products displaying claims related to dietary fibre, iron, and micronutrients (unspecified) doubled in 2021. Claims that emerged in 2021 included protein for longevity (*n* = 3) and optimal health (*n* = 1), and dietary fibre for improved satiety (*n* = 2), while claims in relation to dietary fibre for improved digestive health and bowel function doubled in the last two years (*n* = 3 additional products). The presence of high-level health claims experienced no change over time. Other claims such as ‘vegetarian/vegan’ more than doubled over time, representing the greatest increase since 2019 with an additional 151 products identified, followed by ‘no artificial colours/flavours/preservatives’ (*n* = 140 additional products). ‘Plant-based’ (*n* = 27) and ‘sustainability’ (*n* = 27) claims only emerged in 2021. 

## 4. Discussion

This study aimed to provide an insight into the legume food category and compare nutrition product data and nutrition and health claims obtained in 2019 and 2021. The results demonstrated an increase of 298 legume products over the two years preceding 2021 (*n* = 610), including an increase among all product categories and a relative increase in the variety of legume types available (22.2%), with black-eyed beans, giant beans, great northern beans and lupin making a debut into the market according to our analysis. An increase in product manufacturers (72.7%) suggests a substantial interest within the food industry. The increase in the number of legume products identified by this study is consistent with the trajectory reported by Gilham et al. (2018) [27], who observed an increase of 208 new products with at least half a serve of legumes between 2012 and 2017.

The legume snack category increased 169% compared to 2019, the largest increase among all legume categories, especially legume chips, which increased four-fold. This notable increase demonstrates the innovation within the category, providing consumers the opportunity to obtain dietary legumes via convenient, ready-to-eat snack foods, rather than the more traditional methods such as in soups [27]. While some of these products may not be nutritionally equivalent to their whole food counterparts, several products do show promise as a convenient way to increase legume intake. The legume content of the snack products ranged from 5% to 100% indicating that these products are a means of complimenting overall legume intake.

Legumes are increasingly being recognised as an ecologically sustainable food [9], and the notable emergence of ‘sustainability’ claims featuring on legume products in the two years following 2019 parallels this; however, both the positive health and environmental effects may be offset by the heavy processing required to transform some of these products [28]. In addition to the emergence of on-pack ‘sustainability’ claims, the prevalence of on-pack labeling to identify products as suitable for vegans/vegetarians also increased considerably, demonstrating the largest increase (*n* = 151) compared to all other on-pack claims. In line with the theme of vegan/vegetarianism, food marketing has evolved to appeal to consumer trends. This has seen the emergence of the term ‘plant-based’ used on-pack among legume products in a bid to appeal to consumers. A total of 4.43% of products displayed the term ‘plant-based’ in 2021, with a comparator of zero in 2019. This trend is widespread among the food industry with use of the term among all Australian food product launches increasing by 26.7% over the two years preceding 2021 [29].

As the evidence base for diet-induced modulation of the gut microbiome to improve overall health has grown [30], so too has consumer interest in eating to improve gut health, and this was demonstrated among on-pack claims identified among legume products. Both general-level health claims related to digestive health and nutrition content claims related to dietary fibre doubled over the last two years. As the body of evidence suggesting an association between legume consumption and modulation of the gut microbiota continues to emerge [31,32], it may be expected that on-pack claims of this nature will continue to increase in prominence among legume products. 

This study is the first of its kind, to our knowledge, to comprehensively review legume products available in Australian supermarkets. There are several limitations within the study design that must be acknowledged. While all efforts were made to identify legume products in their entirety, differences in product availability may exist within different geographic locations of supermarkets. Furthermore, the 2021 data collection took place after the global COVID-19 pandemic had commenced, which may have impacted some product availability.

The findings of the research provide insight into the changing landscape of legume products available to Australian consumers. The data obtained by this research may be used as an aid to inform government bodies involved in the reform of national healthy eating guidelines, as it indicates that the scope of dietary legume consumption may no longer fall within traditional culinary classifications of ‘vegetables’ and ‘lean meat and alternatives’, but instead as a distinct food group on its own. Future development of legume consumption surveys should also consider the findings of this research, as variability in nutritional quality among categories of legume foods may present as a complexity when aiming to quantify legume intake, particularly in consumption studies. While this research points to an increase in the legume products available, it is unknown whether this has translated to an increase in legume consumption. To progress research within this area, future studies could include a focus on the consumption patterns of such products.

## 5. Conclusions

The main findings of this research demonstrate that the legume product market within Australia has expanded by 95.5%, including an expansion across all categories, with new and innovative opportunities to increase legume intake. Among these legume products, variability does exist with respect to legume content and nutritional composition. While consumption of whole, minimally processed foods is preferable for both human and planetary health, this research suggests that emerging legume products do have the capacity to offer a means of complementing legume intake and may assist with increasing overall consumption.

## Figures and Tables

**Table 1:** Classification and description of legume product categories.

Category	Description of Categories
Baked beans	Navy/haricot beans canned in tomato sauce with the term ‘baked beans’ in the product name.
Legume dips	Commercial dips derived from cooked, blended legumes, with a type of legume captured in the product name or included as an ingredient, e.g., hummus or black beans.
Legume flours	Flour derived from dried, ground (uncooked) legumes, e.g., chickpeas, red lentils, or soybeans.
Legume snacks	Ready-to-eat packaged snack foods available in the snack food aisle or health food aisle, with a type of legume captured in the product name or included as the main ingredient. Sub-categories include legume chips, derived from legume flour, whole legumes, savoury and whole legumes, sweet.
Canned legumes	Legumes that have been boiled and canned in brine, as well as ready-to-eat legumes that have been boiled, drained, and packaged into pouches, e.g., chickpeas, lentils, kidney beans, peas. Excludes combination products, e.g., tuna and bean mixes.
Dried legumes	Dried and uncooked legumes, e.g., dried split peas, dried chickpeas, or soup mixes with legumes.
Frozen legumes	Commercial frozen legume products, e.g., frozen broad beans, edamame, or peas. Excludes combination products, e.g., corn and peas, ready meals, and meat alternatives.
Pulse pasta	Pasta made with flour derived from dried, ground legumes, e.g., chickpeas, red lentils, or peas.

**Table 2:** The number of legume products identified per category and sub-category in both 2019 and 2021 and the change over time.

Category	2019 *n* (% of Total)	2021 *n* (% of Total)	Change 2019–2021 *n* (%)
Canned legumes	82 (26.3)	154 (25.2)	72 (87.8)
Legume snacks	52 (16.7)	140 (23.0)	88 (169)
*Legume chips*	21 (6.73)	96 (15.7)	75 (357)
*Whole legumes, savoury*	29 (9.29)	37 (6.07)	8 (27.6)
*Whole legumes, sweet*	2 (0.64)	7 (1.15)	5 (250)
Legume dips	76 (24.4)	107 (17.5)	31 (40.8)
Dried legumes	29 (9.29)	92 (15.1)	63 (217)
Baked beans	35 (11.2)	47 (7.70)	12 (34.3)
Frozen legumes	25 (8.01)	32 (5.25)	7 (28.0)
Pulse pasta	11 (3.53)	32 (5.25)	21 (190)
Legume flours	2 (0.64)	6 (0.98)	4 (200)
Total	312	610	298 (95.5)

**Table 3:** Legumes varieties used in legume products in 2019 and 2021 and the change in number of products over time.

Legume Type	2019 *n* (% of Total)	2021 *n* (% of Total)	Change 2019–2021 *n* (%)
Beans	117 (37.5)	217 (35.6)	100 (85.5)
*Adzuki beans*	1 (0.32)	4 (0.66)	3 (300)
*Beans (unspecified)*	1 (0.32)	5 (0.82)	4 (400)
*Beans, mixed* ^a^	9 (2.88)	9 (1.47)	-
*Black beans/Black turtle beans*	12 (3.85)	29 (4.75)	17 (142)
*Black-eyed beans*	-	1 (0.16)	1
*Borlotti beans*	6 (1.92)	14 (2.27)	8 (133)
*Broad beans/Faba (fava) beans*	17 (5.45)	21 (3.44)	4 (23.5)
*Butter beans/Lima beans*	6 (1.92)	9 (1.47)	3 (50.0)
*Cannellini beans*	9 (2.88)	18 (2.95)	9 (100)
*Edamame/Soybeans*	6 (1.92)	24 (3.93)	18 (300)
*Giant beans*	-	1 (0.16)	1
*Great northern beans*	-	2 (0.33)	2
*Haricot beans/Navy beans*	28 (8.97)	37 (6.07)	9 (32.1)
*Mung beans*	1 (0.32)	7 (1.15)	6 (600)
*Pinto beans*	3 (0.49)	5 (0.82)	2 (66.7)
*Kidney beans*	14 (4.49)	29 (4.75)	15 (107)
*White beans (unspecified)*	4 (1.28)	2 (0.33)	−2 (−50.0)
Chickpeas	97 (31.1)	173 (28.4)	76 (78.3)
Peas	48 (15.4)	76 (12.5)	28 (58.3)
Lentils	30 (9.62)	73 (12.0)	43 (143)
Mixed ^b^	20 (6.41)	70 (11.5)	50 (250)
Lupin	-	1 (0.16)	1
Total variety	18	22	4 (22.2)

^a^ A combination of any bean variety listed. ^b^ A combination of any legume type listed.

**Table 4:** The number and proportion of products meeting eligibility criteria for on-pack claims and NPSC in 2021; *n* (% of category).

Nutrition Content Claim	Canned Legumes (*n* = 154)	Legume Snacks (*n* = 140)	Legume Dips (*n* = 107)	Dried Legumes (*n* = 92)	Baked Beans (*n* = 47)	Frozen Legumes (*n* = 32)	Pulse Pasta (*n* = 32)	Legume Flours (*n* = 6)
Low fat (≤3 g per 100 g)	143 (92.6)	2 (1.43)	3 (2.80)	67 (72.8)	46 (97.9)	29 (90.6)	17 (53.1)	1 (16.7)
Low saturated fat (≤1.5 g per 100 g)	145 (94.2)	39 (27.9)	26 (24.3)	90 (97.8)	47 (100)	32 (100)	32 (100)	5 (83.3)
Source of protein (≥5 g per serve)	77 (50.0)	27 (19.3)	8 (7.48)	22 (23.9)	26 (55.3)	15 (46.9)	9 (28.1)	1 (16.7)
Good source of protein (≥10 g per serve)	21 (13.6)	18 (12.9)	2 (1.87)	65 (70.7)	19 (40.4)	1 (3.13)	23 (71.9)	5 (83.3)
Low sodium (≤120 mg per 100 g)	52 (33.8)	10 (7.14)	5 (4.67)	87 (94.6)	3 (6.38)	30 (93.8)	31 (96.9)	6 (100)
Eligible for fibre claim (≥2 g per serve)	136 (100) ^a^	65 (63.1) ^b^	8 (32.0) ^c^	48 (100) ^d^	47 (100)	22 (100) ^e^	30 (100) ^f^	5 (100) ^g^
Source of fibre (≥2–<4 g per serve)	38 (27.9) ^a^	2 (1.94) ^b^	4 (16.0) ^c^	1 (2.08) ^d^	1 (2.13)	6 (27.3) ^e^	4 (13.3) ^f^	0 (0.00) ^g^
Good source of fibre (≥4–<7 g per serve)	66 (48.5) ^a^	20 (19.4) ^b^	2 (8.00) ^c^	22 (45.8) ^d^	16 (34.0)	16 (72.7) ^e^	6 (20.0) ^f^	2 (40.0) ^g^
Excellent source of fibre (≥7 g per serve)	32 (23.5) ^a^	5 (4.85) ^b^	2 (8.00) ^c^	25 (52.1) ^d^	30 (63.8)	0 (0.00) ^e^	20 (66.7) ^f^	3 (60.0) ^g^
Meets NPSC ^h^	154 (100)	73 (52.1)	63 (58.9)	92 (100)	47 (100)	32 (100)	32 (100)	6 (100)

^a^ 136 products reported dietary fibre. ^b^ 103 products reported dietary fibre. ^c^ 25 products reported dietary fibre. ^d^ 48 products reported dietary fibre. ^e^ 22 products reported dietary fibre. ^f^ 30 products reported dietary fibre. ^g^ 5 products reported dietary fibre. ^h^ Nutrient Profiling Scoring Criterion (NPSC). To pass the NPSC, the final score must be <4.

**Table 5:** Frequency of legume products displaying nutrition content and health claims in 2019 and 2021.

	2019 *n* (% of Total)	2021 *n* (% of Total)	Change 2019–2021 *n* (%)
**Nutrition Content Claim**			
Dietary Fibre	118 (37.8)	246 (40.3)	128 (108)
Gluten Free	100 (32.1)	216 (35.4)	116 (116)
Protein	94 (30.1)	208 (34.1)	114 (121)
Fat	68 (21.8)	90 (14.8)	22 (32.4)
Salt	32 (10.3)	56 (9.18)	24 (75.0)
Sugar	8 (2.56)	34 (5.57)	26 (325)
Energy	10 (3.21)	28 (4.59)	18 (180)
Vitamins/Minerals	5 (1.60)	24 (3.93)	19 (380)
Glycemic Index	10 (3.21)	19 (3.11)	9 (90.0)
Carbohydrate	1 (0.32)	15 (2.46)	14 (1400)
General-Level Health Claim			
Protein	3 (0.96)	13 (2.13)	10 (333)
Dietary Fibre	6 (1.92)	12 (1.97)	6 (100)
Iron	1 (0.32)	3 (0.49)	2 (200)
Vitamin C	3 (0.96)	3 (0.49)	-
Micronutrients (unspecified)	1 (0.32)	2 (0.33)	1 (100)
Thiamin (B1)	-	2 (0.33)	2
**High-Level Health Claim**			
F&V; CHD	1 (0.32)	1 (0.16)	-
Saturated fat; CHD	1 (0.32)	1 (0.16)	-
**Other Claims ^a^**			
No Artificial C/F/P ^b^	112 (35.9)	252 (41.3)	140 (125)
Vegetarian/Vegan	81 (26.0)	232 (38.0)	151 (186)
Organic	34 (10.9)	115 (18.8)	81 (238)
Plant-based ^c^	-	27 (4.43)	27
Sustainability	-	27 (4.43)	27

^a^ Claims that are not outlined in Standard 1.2.7 of the Food Standards Code. ^b^ Colours/Flavours/Preservatives (C/F/P). ^c^ Includes specific terms ‘plant-based’, ‘plant protein’ and ‘plant power’. Fruit and Vegetables (F&V); coronary heart disease (CHD).

## Data Availability

All data for this study are contained within the article.
